# Wild Mushroom Extracts as Inhibitors of Bacterial Biofilm Formation

**DOI:** 10.3390/pathogens3030667

**Published:** 2014-08-06

**Authors:** Maria José Alves, Isabel C. F. R. Ferreira, Inês Lourenço, Eduardo Costa, Anabela Martins, Manuela Pintado

**Affiliations:** 1 CBQF-Escola Superior de Biotecnologia, Universidade Católica Portuguesa Porto, Rua Dr. António Bernardino de Almeida, 4200-072 Porto, Portugal; E-Mails: maria.alves@ipb.pt (M.J.A.); emcosta@porto.ucp.pt (E.C.); 2 Centro Hospitalar de Trás-os-Montes e Alto Douro-Unidade de Chaves, Av. Dr. Francisco Sá Carneiro, 5400-249 Chaves, Portugal; 3 Centro de Investigação de Montanha (CIMO), ESA, Instituto Politécnico de Bragança, Campus de Santa Apolónia, Apartado 1172, 5301-855 Bragança, Portugal; E-Mail: amartins@ipb.pt; 4 Escola Superior de Saúde, Instituto Politécnico de Bragança, Av. D. Afonso V, 5300-121 Bragança, Portugal; E-Mail: ines.evamix@gmail.com

**Keywords:** clinical isolates, biofilm, wild mushroom extracts, multi-resistant, cytotoxicity

## Abstract

Microorganisms can colonize a wide variety of medical devices, putting patients in risk for local and systemic infectious complications, including local-site infections, catheter-related bloodstream infections, and endocarditis. These microorganisms are able to grow adhered to almost every surface, forming architecturally complex communities termed biofilms. The use of natural products has been extremely successful in the discovery of new medicine, and mushrooms could be a source of natural antimicrobials. The present study reports the capacity of wild mushroom extracts to inhibit* in vitro* biofilm formation by multi-resistant bacteria. Four Gram-negative bacteria biofilm producers (*Escherichia coli*,* Proteus mirabilis*,* Pseudomonas aeruginosa*, and *Acinetobacter baumannii*) isolated from urine were used to verify the activity of *Russula delica*,* Fistulina hepatica*,* Mycena rosea*,* Leucopaxilus giganteus*, and * Lepista nuda *extracts. The results obtained showed that all tested mushroom extracts presented some extent of inhibition of biofilm production. *Pseudomonas aeruginosa *was the microorganism with the highest capacity of biofilm production, being also the most susceptible to the extracts inhibition capacity (equal or higher than 50%). Among the five tested extracts against *E. coli*, *Leucopaxillus giganteus* (47.8%) and *Mycenas rosea* (44.8%) presented the highest inhibition of biofilm formation. The extracts exhibiting the highest inhibitory effect upon *P. mirabilis* biofilm formation were *Sarcodon imbricatus* (45.4%) and *Russula delica* (53.1%). *Acinetobacter baumannii *was the microorganism with the lowest susceptibility to mushroom extracts inhibitory effect on biofilm production (highest inhibition—almost 29%, by *Russula delica* extract). This is a pioneer study since, as far as we know, there are no reports on the inhibition of biofilm production by the studied mushroom extracts and in particular against multi-resistant clinical isolates; nevertheless, other studies are required to elucidate the mechanism of action.

## 1. Introduction

Antimicrobial resistance (AMR) is a serious threat to public health. The level of AMR, especially multidrug resistance, is increasing in Europe, leading to high healthcare costs associated with high morbidity and mortality levels [1].

Multi-resistance is considered to be a key indicator of problematic bacterial strains because it constrains empirical treatment regimens and reduce the options of appropriate treatments [2]. This situation is considered to be an infection control priority to manage patient mortality and limit the spread of multi-resistant strains.

Microorganisms can colonize a wide variety of medical devices, putting patients in risk for local and systemic infectious complications, including local-site infections, catheter-related bloodstream infections, and endocarditis [3]. Bacteria, such as *Escherichia coli*, *Pseudomonas aeruginosa*, *Klebsiella pneumoniae*, *Proteus mirabilis*, *MRSA* (methicillin-resistant* Staphylococcus aureus*) and *Enterococcus *spp., are involved in urinary infections with the highest biofilm production rates [4,5,6,7,8]. According to the Annual Epidemiological Report of 2013, *Acinetobacter baumannii *also presents, in Portugal, a higher prevalence (4.5%) than the European Union medium (1.9%) in urinary infections of catheterized patients in Health Intensive Care Unities [1].

These bacteria are able to grow adhered to almost every surface, forming architecturally complex communities termed biofilms [9,10,11,12]. Encased in a complex polysaccharide matrix produced by the bacteria themselves, a biofilm permits to protect the bacteria against antibiotics, being the cause of many recalcitrant infections.

In the last decade, several strategies to control biofilm growth on medical devices have been suggested, including the use of topical antimicrobial ointments, minimizing the length of time of catheterization, using catheters provided with a surgically implanted cuff [13], and coating the catheter lumen with antimicrobial agents [14,15,16,17,18,19,20,21,22]. However, these antimicrobial-loaded catheters may pose several limitations, including the rapid release of the adsorbed antibiotic in the first hours after implantation and, as a result, a relatively short persistence of antibacterial action [23]. The risk of emerging multidrug-resistant pathogens is continuously growing due to the extensive use of antibiotics both in prophylaxis and long-term therapy. Microorganisms growing in a biofilm are much more resistant to antimicrobial agents than planktonic cells and hence treatment of biofilm contaminated surfaces with conventional antimicrobials may fail as it is known that it takes >1000 times more antibiotics to kill biofilm cells than to kill planktonic cells [9].

The use of natural products has been extremely successful in the discovery of new medicine [24]. According to Harvey [24], the access to biodiversity is fundamental to expanding the range of natural products to be used in the search for new drugs. In this context, mushrooms, which remain unexplored in this area, might be a valuable resource in the search of new bioactive extracts/compounds to inhibit biofilm production.

In fact, mushrooms could be a source of natural antibiotics. The extracts of some species, including *Laetiporus sulphureus* [25], *Ganoderma lucidum* [26], and *Lentinus edodes* [27] have already demonstrated antibacterial activity. Furthermore, Alves * et al.* [28], demonstrated that *Fistulina hepatica*, *Ramaria botrytis*, and *Russula delica* extracts were promising against multi-resistant microorganisms namely MRSA, *Escherichia coli* and *Proteus mirabilis*. This activity was mainly attributed to phenolic acids present in the methanol: water extracts [29]. Additionally, Jagani* et al.* [30] have shown that phenol and natural phenolic compounds cause a significant reduction in biofilm formation by *P. aeruginosa*. Soković* et al.* [31] described anti quorum sensing activity of *Agaricus blazei* hot water extract in the same bacteria. In addition, the effects of *Lentinus edodes* extract and purified fractions on human oral pathogens in biofilm state have been described [32].

Therefore, the present study reports the capacity of wild mushroom metanol:water extracts to inhibit* in vitro* biofilm formation in multi-resistant bacteria isolated from clinical specimens. Being the mentioned extracts rich in phenolic compounds [29], these natural molecules can provide an excellent alternative for inhibition of biofilm production. Furthermore, the toxicity of the tested extracts was also assessed in order to guarantee the safety of their use. As far as we know, this is the first study on the inhibition of biofilm production by the tested mushroom extracts and in particular against multi-resistant clinical isolates.

## 2. Results and Discussion 

Data available in Table 1 show that, in general, all tested mushroom extracts presented some extent of inhibition of biofilm production according the different tested microorganisms. Furthermore, none of the extracts, at the tested concentrations (up to 20 mg/mL), have shown toxicity against a primary culture of porcine liver cells (PLP2; the GI_50_- concentration responsible for inhibition of 50% of the cells net growth- of the positive control, ellipticine was 3.8 ± 0.5 μM).

**Table 1:** Effect of sub-MIC (minimal inhibitory concentrations) of wild mushroom extracts in the biofilm production by different clinical isolates (Mean ± SD; *n* = 3).

Mushroom	Average Production Inhibition of Biofilm (%)			
	*P. aeruginosa *	*E. coli*	*A. baumannii*	*P. mirabilis *
***L. nuda***	55.99 ± 1.13 ª	22.89 ± 1.53 ^c^	14.22 ± 2.52 ^b^	39.73 ± 2.17 ^b^
***L. giganteus ***	56.79 ± 8.78 ª	47.84 ± 0.93 ª	15.12 ± 0.58 ^b^	41.72 ± 6.53 ^ab^
***M. rosea ***	53.36 ± 8.02 ª	44.88 ± 0.68 ª	16.08 ± 1.05 ^b^	40.24 ± 2.08 ^ab^
***R. delica***	57.35 ± 5.01 ª	29.37 ± 4.95 ^bc^	28.59 ± 0.70 ª	60.31 ± 1.20 ^a^
***S. imbricatus ***	57.49 ± 6.63 ª	32.41 ± 5.86 ^b^	17.15 ± 1.97 ^b^	49.97 ± 2.53 ^ab^

In each column different letters mean significant differences (*p* < 0.05).

**Figure 1:**
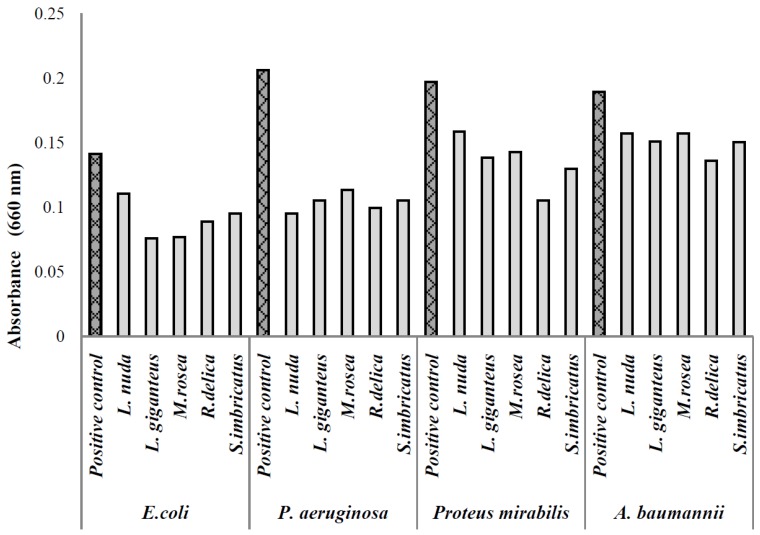
Absorbance values of the biofilm formed in the presence of various mushroom extracts.

**Figure 2:**
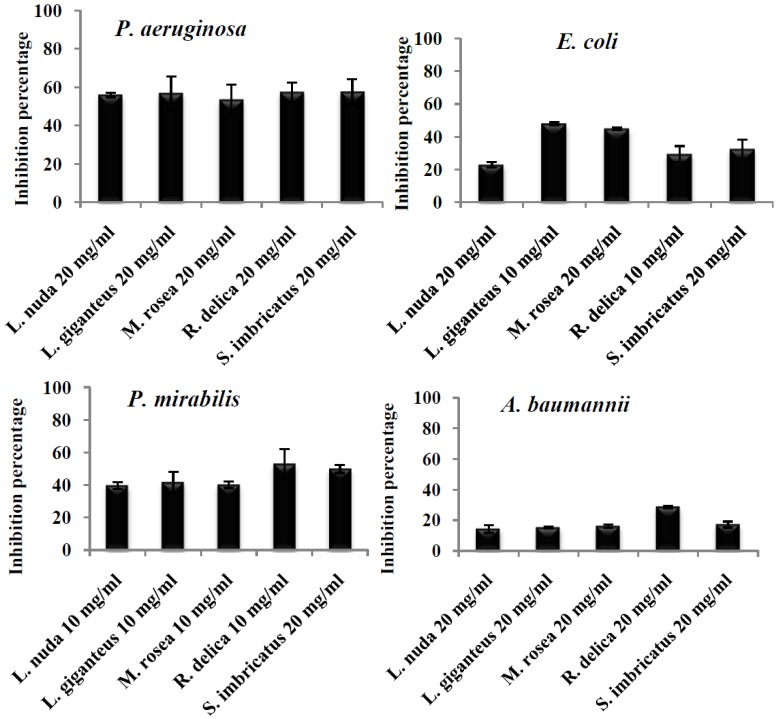
Percentage of inhibition of biofilm production exerted by mushroom extracts upon different clinical isolates.

*Pseudomonas aeruginosa *(resistant to cephalosporins, amoxicillin/clavulanic acid, fluoroquinolonas and trimethoprim sulfaxonazol) was the microorganism with highest capacity of biofilm production (Figure 1) and was the microorganism that exhibited the highest inhibition percentage (equal or higher than 50%) of biofilm formation by the different extracts at the tested concentration, without significant statistical differences (*p* < 0.05) (Figure 2).

Other authors [30,33,34] also reported excellent results of different pure compounds present in natural products (usnic acid, terpenes and various phenolic compounds) in the inhibition of biofilm production by *P. aeruginosa. *Particularly, Jagani* et al.* [30] demonstrated the potential of phenol (80.9%) and natural phenolic compounds (e.g., tannic acid—78.1%, eppigallocatechin—69.9%, catechin—67.5%) to inhibit biofilm of the mentioned bacteria.

In addition, Borges* et al.* [35] demonstrated that flavonoids (ferulic and gallic acid) inhibited the ability of biofilm production in *P.* *aeruginosa*, fact that the authors related to the interference in cell motility and physico-chemical changes in the cell surface.

Considering that the extracts tested in the present work possess phenolic compounds (e.g., phenolic acids and a few flavonoids, Table 2), these molecules could be responsible for the observed properties. Moreover, it should be highlighted that these results are very relevant since *P. aeruginosa* is one of the problematic microorganisms in hospital environment, being highly involved in infections related with health care (HAI) due to multi-resistance and high capacity to produce biofilm. According to Annual Epidemiological Report of 2013 [1], *P. aeruginosa *(12.5%) is among the five microorganisms most isolated from acquired urinary tract infections in patients of European Health Intensive Care Unities. Furthermore, this bacterium is cited for its high resistance to different antibiotic groups (aminoglycosides, fluoroquinolones and carbapenems [1], also observed for the clinical isolate used herein for fluoroquinolones.

According to Hancock* et al.* [36], ellagic acid and tannic acid potentiate the action of the substance thioridazine known as an inhibitor of efflux pumps, producing a greater anti-biofilm activity in *E. coli*.

Vikram* et al.* [37], tested the capacity of flavonoids to inhibit biofilm of *E. coli* O157:H7, and quercetin showed to be the most potent one. Nevertheless, flavonoids are not commonly found in mushrooms, being phenolic acids the most abundant phenolic compounds [38]; Table 2). Borges* et al.* [35] found that ferulic and gallic acids showed inhibitory capacity on the production of biofilms in *E. coli*; probably the results obtained for *E. coli* in the present work (Table 1) might be due to the presence of phenolic compounds in the tested extracts (Table 2), but also other non-identified compounds (see for example *Leucopaxillus giganteus *results in Table 2). Among the five tested extracts against *E. coli*, *Leucopaxillus giganteus* (47.8%), and *Mycenas rosea* (44.8%) presented the highest significant (*p* < 0.05) biofilm inhibition. Another important aspect is that the *E. coli *strain used in the present work is a clinical isolate and not a collection microorganism as the one used by Vikram* et al.* [37]. The extracts exhibiting the best inhibitory effect upon *P. mirabilis *(resistant to fluoroquinolones, ampicillin and cephalosporins) biofilm formation were *Sarcodon imbricatus *(45.4%) and *Russula delica* (53.1%). In fact, the inhibition effect of the tested mushroom extracts upon *Proteus mirabilis *was lower than the ones obtained for *P. aeruginosa* (Figure 2), but still relevant, due to the high capacity of *P. mirabilis* to produce biofilm in urinary catheters an due to its high antibiotic resistance. Some microorganisms such as *P. mirabilis* change pH values by producing urease, which hydrolyses urea to ammonia that increases pH value, promoting the precipitation of minerals, which in turn are deposited on catheters causing mineral inlays and stimulating biofilms production [39]. No study was found reporting the activity of pure phenolic compounds or natural extracts on biofilm formation.

*Acinetobacter baumannii* was the microorganism with the lowest susceptibility to mushroom extracts inhibitory effect on biofilm production (Figure 2); *Russula delica* extract inhibited biofilm production by almost 29%, showing the best performance among all the extracts (*p* < 0.05) (Figure 1). Being this microorganism extremely resistant to different groups of commercial antibiotics, results obtained are promising in the control of biofilm production. The lower capacity of inhibition of biofilm production by this microorganism might be related with the highest resistance profile of *Acinetobacter* spp. to different antibiotic groups namely, carbapenems (81.2%), observed in Europe [1]. In particular, the strain used in the present work is resistant to β-lactamic antibiotics (ampicillin cephalosporins, amoxicillin/clavulanic acid) and fosfomycin.

This is a pioneer study since, as far as we know, there are no reports on the inhibition of biofilm production by the studied mushroom extracts and in particular against multi-resistant clinical isolates involved in the study; nevertheless, other studies are required to elucidate the mechanism of action. Concerning the mechanism of action of phenolic on the inhibition of biofilm, although scarce information is available some authors [40,41,42] demonstrated that phenolic acids may possibly interferer with Quorum sensing inhibiting the process of biofilm formation. Sokovic* et al.* [31], also reported that hot water extract from *Agaricus blazei* could influence on biofilm formation, twitching and swimming activity, pyocianin production which are part of anti quorum sensing activity.

**Table 2:** Total phenolic content determined by Folin Ciocalteu-assay and individual phenolic compounds identified by HPLC-DAD (high performance liquid chromatography-diode array detection) in the tested wild mushroom extracts (Mean ± SD; *n* = 3).

Mushroom	Phenolics (mg GAE/g MWE Extract)	Protocatechuic Acid (mg/kg dw)	*p*-Hydroxybenzoic Acid (mg/kg dw)	*p*-Coumaric Acid (mg/kg dw)	Gallic acid (mg/mL EE)	Caffeic Acid (mg/mL EE)	Catechin (mg/mL EE)	Rutin (mg/mL EE)
***L. nuda*^1,2^**	6.31 ± 0.13 ^a^	33.47 ± 0.50	29.31 ± 1.54 ^b^	3.75 ± 0.56	-	-		-
***L. giganteus*^3^**	6.29 ± 0.20 ^a^	-	-	-	-	-		-
***M. rosea*^3^**	3.56 ± 0.37 ^c^	na	na	na	na	na		na
***R. delica*^3,4^**	2.23 ± 0.18 ^d^	-	-	-	0.05	0.11	5.33	0.46
***S. imbricatus*^1^**	3.76 ± 0.11 ^b^	-	33.19 ± 1.92 ^a^	-	-	-		-

^1^ [43]; ^2^ [44]; ^3^ [45]; ^4^ [46]. GAE—gallic acid equivalentes; MWE—metanol:water extract; EE—ethanolic extract; dw—dry weight; na—not available. In each column different letters mean significant differences (*p* < 0.05).

Overall, the use of biomaterials in several biotechnological applications such as prosthesis, implants, urinary and venous catheters, is common in the clinical practice. Otherwise, these biomaterials are often colonized by pathogenic microorganisms, which can lead to serious infections and even rejection. Therefore, the search and development of new antimicrobial and especially antibiofilm solutions to coat these biomaterials conferring them the capacity to avoid the development of these microorganisms is crucial. However, the safety of these extracts must be proved to guarantee their safe application as antimicrobial or antibiofilm.

Accordingly, and considering the absence of cytotoxicity of the studied mushroom extracts (tested in primary cultures of porcine liver cells), they can be safely incorporated in biomaterials used in catheters, prosthesis and other medical devices in order to avoid microorganism adhesion and biofilm production.

## 3. Materials and Methods

### 3.1. Extracts Preparation

Each mushroom lyophilized sample (*ca.* 3 g) was extracted using a methanol:water (80:20; 30 mL) mixture at −20 °C for 6 h. After 15 min in an ultrasonic bath, the extract was centrifuged at 4000× *g* for 10 min and filtered through Whatman n° 4 paper. The residue was then extracted with two additional 30 mL portions of the methanol:water mixture. The combined extracts were evaporated at 40 °C under reduced pressure to remove methanol (rotary evaporator Büchi R-210, Flawil, Switzerland), lyophilized, redissolved in water, at a concentration of 200 mg/mL, and stored at −20 °C for further use.

### 3.2. Bacterial Isolates

The microorganisms used were the major clinical isolates from patients hospitalized in various departments of the Hospital Center of Trás-os-Montes and Alto-Douro, Chaves, Portugal. Thus, four Gram-negative bacteria biofilm producers (*Escherichia coli*,* Proteus mirabilis*,* Pseudomonas aeruginosa* and *Acinetobacter baumannii)* isolated from urine were used to verify that extracts of *Russula delica*,* Fistulina hepatica*,* Mycena rosea*,* Leucopaxilus giganteus *and* Lepista nuda * have the ability to inhibit biofilm formation.

In the case where MIC where found in previous work [28] for the tested extracts (*E. coli*,* P. mirabilis*) a sub-MIC concentration (half of MIC concentration) was used. In the cases where the MICs are not available, the maximal extract concentration (20 mg/mL) was tested, including: all the extracts against *P. aeruginosa* and *A. baumannii; Russula delica *and *Fistulina hepatica* against *P. mirabilis*; and *Lepista nuda *and* Mycena rosea * against *E. coli*.

The isolation and characterization of strains, duly approved by the Ethics Committee, was conducted in the CHTMAD (Hospital Center of Trás-os-Montes and Alto-Douro, Chaves, Portugal), which is a public institution with 182 beds located in Chaves, North of Portugal.

### 3.3. Isolates Identification and Antimicrobial Susceptibility Testing

Microorganism’s identification and susceptibility tests were performed using MicroScan panels (MicroScan^®^; Siemens Medical Solutions Diagnostics, West Sacramento, CA, USA) by microdilution plate method. The interpretation criteria were based on Interpretive Breakpoints as indicated in Clinical and Laboratory Standards Institute (CLSI) Document M100-S18 [47] and the report of the *Committee of L’Antibiogramme de la Société Française de Microbiologie *(CA-SFM) [48].

### 3.4. Inhibition of Biofilm Formation

Quantification of biofilm production was carried out by adapting the microtiter biofilm formation protocol described by Stepanovic* et al.* [49]. Briefly, in a flat bottom 96 microplate, wells were filled with 200 μL of test solutions at sub-MIC concentrations (or 20 mg/mL, according section 2.2) with inoculum being added at 2% (v/v). Following this the microplate was incubated at 37 °C for 48 h. To visualize biofilms, the contents of each well were discarded and the well washed 3 times with sterile deionized water in order to remove non-adherent cells. The remaining attached bacteria were fixed with 200 μL of ethanol (Panreac, Barcelona, Spain) for 15 min. Ethanol was then discarded and the wells air dried. After that, 200 μL of crystal violet solution (Merck, Darmstadt, Germany) were added to the wells for 5 min. Excess stain was removed by rinsing the plate under tap water and the air dried.

Adherence was quantified by measuring the Optical Density (OD) at 660 nm using a microplate reader (FlUOstar, OPTIMA, BGM Labtech, Ortenberg, Germany).

Results for this test were given as percentage of biofilm formation inhibition applying the following formula:

*Biofilm formation inhibition percentage = 100 *−* (OD_assay_*/*OD_control_) x 100* (1)

All assays were done in triplicate.

### 3.5. Toxicity Assay

A cell culture was prepared from a freshly harvested porcine liver obtained from a local slaughter house, and it was designed as PLP2. Briefly, the liver tissues were rinsed in Hank’s balanced salt solution containing 100 U/mL penicillin, 100 µg/mL streptomycin and divided into 1 × 1 mm^3^ explants. Some of these explants were placed in 25 cm^2^ tissue flasks in DMEM medium supplemented with 10% fetal bovine serum, 2 mM nonessential amino acids and 100 U/mL penicillin, 100 mg/mL streptomycin and incubated at 37 °C with a humidified atmosphere containing 5% CO_2_. The medium was changed every two days. Cultivation of the cells was continued with direct monitoring every two to three days using a phase contrast microscope. Before confluence, cells were subcultured and plated in 96-well plates at a density of 1.0 × 10^4^ cells/well, and cultivated in DMEM medium with 10% FBS, 100 U/mL penicillin and 100 μg/mL streptomycin. Cells were treated for 48 h with the tested extracts and the sulforhodamine B (SRB) colorimetric assay was performed. Briefly, cells were fixed by adding cold 50% (w/v) trichloroacetic acid (TCA, 25 μL) and incubated for 60 min at 4 °C. Plates were then washed with deionized water and dried; SRB solution (0.1% w/v in 1% acetic acid, 50 μL) was then added to each plate well and incubated for 30 min at room temperature. Unbound SRB was removed by washing with 1% acetic acid. Plates were air-dried and bound stain was solubilized with 100 μL of a 100 mM Tris base solution. Optical densities were read on an automated spectrophotometer plate reader at a single wavelength of 540 nm (Biotek Elx800, Winooski, VT, USA). Ellipticine was used as positive control at a concentration of 5 μM [50].

### 3.6. Statistical Analysis

Three samples were used for each species and tested bacteria and all the assays were carried out in triplicate for both antibiofilm activity and cell toxicity. The results are expressed as mean values and standard deviation (SD). The results were analyzed using one-way analysis of variance (ANOVA) followed by Tukey’s HSD Test with α = 0.05. This treatment was carried out using SPSS v. 22.0 program (IBM Corp., USA).

## 4. Conclusions

The studied mushroom extracts present, in general, good capacity to inhibit biofilm production. Among the tested microorganisms, *P. aeruginosa* seems to be the most susceptible one to the antibiofilm activity of the mushroom extracts. It should be highlighted that in some cases where the extract concentrations were unable to completely inhibit bacterial growth, the antibiofilm effect was observed.

Considering that the tested bacteria were clinical isolates with high antibiotic resistance profile, the promising results obtained are indeed relevant, so the mechanism of action involved should be further studied, in order to clarify the role of mushroom extracts in this dual problematic: multi-resistance and high biofilm production.
